# Birth Asphyxia among Neonates Admitted to the Neonatal Intensive Care Unit of a Tertiary Care Hospital

**DOI:** 10.31729/jnma.8429

**Published:** 2024-02-29

**Authors:** Dhirendra Prasad Yadav, Vivek Kumar, Manoj Kumar Gupta

**Affiliations:** 1 Department of Pediatrics, National Medical College Teaching Hospital, Birgunj, Parsa, Nepal; 2 Department of Pediatrics, All India Institute of Medical Sciences, Ansari Nagar, New Delhi, India

**Keywords:** *hypoxic-ischemic encephalopathy*, *neonates*, *prevalence*

## Abstract

**Introduction:**

Birth asphyxia causes significant morbidity and mortality among neonates, especially in low-income and middle-income countries like Nepal. However, there is a paucity of data regarding its burden. This study aimed to find the prevalence of birth asphyxia among neonates admitted to the neonatal intensive care unit of a tertiary care hospital.

**Methods:**

This descriptive cross-sectional study was conducted among neonates at a tertiary care hospital between 15 January 2022 to 14 January 2023 after obtaining ethical approval from the Institutional Review Committee. Neonates with gestational age >35 weeks were included and those with major congenital anomalies were excluded. A convenience sampling method was used. A point estimate was calculated at a 95% Confidence Interval.

**Results:**

Among 902 neonates, 120 (13.30%) (11.08-15.52, 95% Confidence Interval) had birth asphyxia. A total of 108 (90%) were outborn, and 84 (70%) were males. HIE stage-I, II and III were seen in 47 (39.17%), 64 (53.33%) and 9 (7.50%) of the asphyxiated neonates respectively. Poor suck 92 (76.67%), seizures 73 (60.83%) and lethargy 70 (58.33%) were common abnormal neurological findings. Death occurred in 15 (12.50%) neonates in the hospital.

**Conclusions:**

The prevalence of birth asphyxia was found to be similar to other studies done in similar settings. The high burden underscores an urgent need to implement better perinatal care and delivery room management practices.

## INTRODUCTION

Birth asphyxia is one of the most common causes of neonatal and under-5 mortality globally. Defined by the World Health Organization as 'the failure to initiate and sustain breathing at birth,^1^ it poses an even greater challenge in low- and middle-income countries like Nepal due to lack of resources and trained manpower.

Asphyxial injury may involve any organ system but hypoxic ischemic encephalopathy (HIE) is the most serious sequelae clinically.^2^ Sarnat and Sarnat proposed a useful clinical tool to assess severity among suspected HIE neonates.^3^ Longterm outcomes of birth asphyxia include permanent neurodevelopmental abnormalities like cerebral palsy, epilepsy and intellectual disability.^4^ Thus, the impact of birth asphyxia is not only limited to clinical morbidity and mortality, but it also adds significantly to the socioeconomic and psychological burden for families.

This study aimed to find the prevalence of birth asphyxia among neonates admitted to the neonatal intensive care unit of a tertiary care hospital.

## METHODS

This descriptive cross-sectional study was conducted at the Neonatal Intensive Care Unit (NICU) of the Department of Pediatrics, National Medical College Teaching Hospital, Birgunj, Nepal from 15 January 2022 to 14 January 2023 after obtaining ethical approval from the Institutional Review Committee (Reference number: F-NMC/551/078-079). Neonates with gestational age ≥35 weeks admitted to the NICU were included in the study. Neonates with major congenital anomalies were excluded from the study. Only ≥35 weeks neonates were included since preterm neonates are more susceptible to ischemia due to incomplete blood-brain barrier and therapeutic hypothermia is recommended for neonates ≥35 weeks and we wanted to assess the prevalence of asphyxia among this gestation category. A convenience sampling method was used. The sample size was calculated by using the following formula:


$$\begin{array}{ll} \text{n} & ={\text{Z}}^{2}\times \frac{\text{p}\times \text{q}}{{\text{e}}^{\text{2}}}\\ & =1.96^{2}\times \frac{0.088\times 0.912}{0.02^{2}}\\ & =771 \end{array}$$

Where,

n = minimum required sample size
Z = 1.96 at a 95% confidence interval
p = prevalence of birth asphyxia from the previous study, 8.8%^5^
q = i-p
e = margin of error, 2%

The calculated sample size was 771. However, 902 neonates were included in our study.

Written informed consent was taken from the parents before enrollment in the patient consent form. Birth asphyxia was considered if neonates (a) presented with a history of delayed or absent cry at birth (as reported by parents or written in referral summary), (b) needed positive pressure ventilation for one minute, or (c) had APGAR score of less than seven at one minute. The NICU is a 20-bed level III with ventilation facilities. However, therapeutic hypothermia is not available at the unit. After enrolment, the details regarding the place of birth, age at admission, gestational age, birth weight, gender, mode of delivery, APGAR score if recorded, method and duration of resuscitation if recorded, maternal age, gravid number, maternal perinatal risk factors were recorded in a predesigned proforma for asphyxiated neonates. The neonates underwent a detailed neurological examination by a trained resident/paediatrician within 2 hours of admission, at 24±2, 48±4 and 72±6 hours. Abnormal neurological findings within the first 24 hours of admission were recorded. The staging of hypoxic-ischemic encephalopathy (HIE) was assessed according to Sarnat and Sarnat's staging at each examination.^3^ The worst stage within 72 hours was assigned as the final HIE stage for the neonate. All the neonates were managed as per unit protocol. No neonates underwent therapeutic hypothermia. Details of hospital course and outcomes were also recorded at the time of discharge/death.

Data were entered in Microsoft Access 2007 and analysed using STATA version 15.1. The point estimate was calculated at a 95% CI.

## RESULTS

Among 902 neonates, the prevalence of birth asphyxia was 120 (13.30%) (11.08-15.52, 95% CI) neonates. Among them, 84(70%) of the asphyxiated neonates were male. A total of 108 (90%) neonates were outborn, of which 60 (55.55%) were admitted on day 1 of life (Table 1).

**Table 1:** Baseline characteristics of mothers and neonates (n = 120).

Maternal characteristics	n (%)
**Maternal age (years)**	
< 20	14 (11.67)
20-24	72 (60)
25-29	29 (24.17)
≥ 30	5 (4.17)
**Gravida**	
Primigravida	77 (64.17)
Multigravida	43 (35.83)
**Place of delivery**	
Home	12 (10)
Primary health centre	12 (10)
Hospital	96 (80)
**Mode of delivery**	
Vaginal	88 (73.33)
Instrumental (vaginal)	6 (5)
Cesarean section	26 (21.67)
**Neonatal characteristics**	
**Birth weight (grams)**	
<2500	30 (25)
≥2500	90 (75)
**Gestational age (weeks)**	
35-36	14 (11.67)
37-41	102 (85)
>41	4 (3.33)
**Gender**	
Male	84 (70)
Female	36 (30)
**Place of birth**	
Inborn	12 (10)
Outborn	108 (90)
**Age at admission**	
1 day	72 (60)
2-3 days	37 (30.83)
> 3 days	11 (9.17)
Meconium stained liquor	28 (23.33)
**Method of resuscitation**	
Bag and mask ventilation	49 (40.83)
Intubation	3 (2.50)
Not known	68 (56.67)

The most common neurological dysfunction observed was poor suck in 92 (76.67%) neonates (Table 2).

**Table 2:** Common abnormal neurological findings were observed within 24 hours of admission (n = 120).

Neurological finding	n (%)
Poor suck	92 (76.67)
Seizure	73 (60.83)
Lethargy	70 (58.33)
Abnormal muscle tone	61 (50.83)
Abnormal cry	44 (36.67)
Jitteriness	23 (19.17)

There were 47 (39.17%) cases of birth asphyxia with HIE stage I, 64 (53.33%) cases with HIE stage II and 9 (7.50%) cases with HIE stage III (Table 3). The combined adverse outcome of death or leave against medical advice was seen in 8 (6.67%) neonates in HIE stage III. Seizure was seen in 73 (60.83%) of the neonates, 29 (24.17%) of whom required two or more two anti-epileptic drugs to control seizure.

**Table 3:** Outcomes of neonates with birth asphyxia (n = 120).

Characteristics	n (%)
**HIE stage**	
Stage I	47 (39.17)
Stage II	64 (53.33)
Stage III	9 (7.50)
**Hospital outcome**	
Discharged	83 (69.17)
LAMA	22(18.33)
Death	15(12.50)
Mechanical ventilation	33 (27.50)
Seizure	73 (60.83)
**Number of antiepileptic drugs**	
1	44 (36.67)
2	2218.33)
>2	7(5.83)
**Duration of hospital stay (day)**	
1	10(8.33)
2-7	83 (69.17)
>7	27 (22.50)

## DISCUSSION

In this study, birth asphyxia was seen in 120 (13.30%) neonates at our tertiary care centre. The prevalence of birth asphyxia seen in our study is comparable to the prevalence seen in similar studies conducted in Nepal (14-15.9%).^6^-^7^ However, other studies from Nepal have reported the prevalence varying from 3.6% to 19.3%.^8-11^ The variations in prevalence seen might be due to different definitions used to define birth asphyxia including different populations studied as well as different levels of maternal and perinatal care provided across various centres. We used the WHO definition of birth asphyxia in this study since most of population was outborn with the unavailability of birth resuscitation details. Thus, the prevalence of asphyxia has not decreased substantially over the years despite advancements in neonatal care.

In this study, 108 (90%) neonates were outborn which is higher than the studies done by others (31.7-56%>).^10,11^ This is probably because our centre is a tertiary care referral centre in that region. This may also point to better antenatal and perinatal care at our centre. A total of 72 (60.00%) of the neonatal admissions in this study were on the first day of birth which is lower in comparison to other studies (90.8-92.8%).^5,10^ This emphasizes the fact that most neonatal problems occur within the first day of life requiring admission. In our study, 49 (40.83%) neonates required bag and mask ventilation. However, the the lack of resuscitation details in 68 (56.67%) of the cases is concerning. This may be due to either no resuscitation being provided, poor birth resuscitation documentation and/or lack of parental counselling.

In this study, 77 (64.17%) mothers were primigravida which is comparable to a previous study (58.82%).^6^ In other studies done in Nepal, the rate of primigravida varied between 37.60-55.00%.^7,9,10^ In our study meconium-stained liquor was reported only in 28 (23.33%) neonates which is comparable to other studies of Nepal (28.00-34.10%).^8,11^ However this finding was lower than other studies reported from Nepal (36.70-69.96%).^6,7,9^ This is important since neonates with meconium-stained liquor have a 7.9 times higher risk of developing birth asphyxia.^12^

In our study, 64 (53.33%) neonates were diagnosed as HIE II followed by 47 (39.17%) HIE I and 9 (7.50%) HIE III. This finding is in contrast to other studies from Nepal which reported a significantly higher number of of neonates with HIE I (65.00-75.49%).^6,7,9^ This difference may be due to most neonates being outborn, thereby having referral bias. Relatively simpler HIE I cases might have been observed and managed at the delivery places. In our study, mortality was 15 (12.50%) which is similar to studies done in Nepal (10.97-15.67%).^6,11^

An interesting observation in our study was that 84 (70%) neonates were male. Since most of our neonates were referred, this may indicate an existing gender bias in favour of males for seeking treatment. Few other studies from Nepal have also reported similar findings (62.30-70.00%).^8,10,11^ This may be due to prevailing social norms and practices. Further studies should explore these possibilities.

However, we acknowledge a few limitations of our study. Firstly, this is a single-centre study with a limited sample size, hence the results may not be generalized to larger settings. Secondly, we could not analyse the risk factors for birth asphyxia as a large number of cases were referred from peripheral hospitals without proper documentation, making it difficult to collect all relevant data. Thirdly, since most of the neonates in our study were referred to, this may have had some selection bias and thus overestimated the true prevalence. Finally, the neonates in our center did not receive therapeutic hypothermia which may have altered the outcomes for the neonates.

## CONCLUSIONS

The prevalence of birth asphyxia among admitted neonates was similar to other studies done in similar settings. The high burden underscores an urgent need to implement better perinatal care and delivery room management practices. Further, larger studies with robust perinatal data are needed to understand the risk factors for birth asphyxia to design appropriate preventive strategies.
